# A comparative study of high-pressure behaviors of the two polymorphs of Ho_2_Ge_2_O_7_

**DOI:** 10.1039/c9ra10428c

**Published:** 2020-03-12

**Authors:** Hui Li, Nana Li, Pinwen Zhu, Xin Wang

**Affiliations:** College of Science, Guangxi University for Nationalities Nanning 530006 China; Center for High Pressure Science and Technology Advanced Research Shanghai 201203 China; State Key Laboratory of Superhard Materials, Jilin University Changchun 130012 China xin_wang@jlu.edu.cn zhupw@jlu.edu.cn; College of Physics, Jilin University Changchun 130012 China

## Abstract

Two polymorphs of polycrystalline Ho_2_Ge_2_O_7_, one with tetragonal structure and the other with cubic structure, were synthesized by using different methods. The structural stabilities of these two polymorphs under high pressure were investigated by angle-dispersive X-ray diffraction (ADXRD). Pressure-induced amorphization was found in the tetragonal Ho_2_Ge_2_O_7_, which is suggested to be associated with the breaking-up of long chains of the edge-shared polyhedron group Ho_4_O_20_. By contrast, cubic Ho_2_Ge_2_O_7_ is stable at high pressures up to 33.3 GPa.

## Abstract 2

Two polymorphs of Ho_2_Ge_2_O_7_ were synthesized using different methods. The structural stabilities were investigated by ADXRD at high pressures.

## Graphical abstract

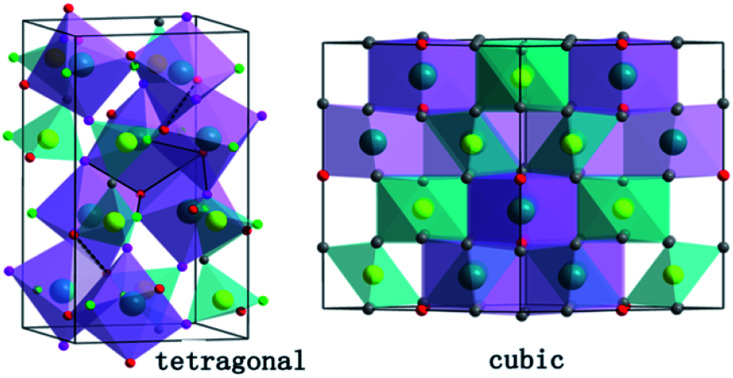

## 1. Introduction

Spin ice has proven to be one of the most fruitful marriages of theoretical and experimental condensed matter physics.^1–4^ It is a remarkable magnetic ground state that can arise in geometrically frustrated pyrochlores, A_2_B_2_O_7_, when magnetic rare-earth ions are situated on the vertices of a lattice of corner-sharing tetrahedra. Competing nearest-neighbor and long-range dipolar interactions result in a short-range ordered ground state for each tetrahedron in which two spins point in and two spins point out.^5^ The spin-ice state was first observed in Ho_2_Ti_2_O_7_ by Harris *et al.* in 1997;^6^ since that time, spin ices have been a subject of active experimentation, allowing theorists to come a long way towards understanding this remarkable ground state. Despite significant interest in this class of compounds, only a handful of spin-ice materials have been discovered to date, including the titanates A_2_Ti_2_O_7_,^6–8^ the stannates A_2_Sn_2_O_7_ ^9,10^ and, more recently, the germanates A_2_Ge_2_O_7_.^11,12^ Recently, the observation of emergent monopole excitations which have captured the attention of the broader scientific community have made spin-ice materials more intriguing.^13–18^ Ho_2_Ge_2_O_7_, a member of the rare-earth pyrogermanate series A_2_Ge_2_O_7_, has been attracting extensive interest because it was found to be a new highly correlated spin-ice material with the highest density of monopoles in the Ho series at low temperatures, and the best natural candidate for monopole studies.^19^

From a structural point of view, rare-earth pyrogermanates A_2_Ge_2_O_7_ possess a variety of crystal structures under ambient conditions, such as triclinic phase for Ln = La, Pr, Nd–Gd, tetragonal phase for Ln = Gd–Lu, and monoclinic phase for In_2_Ge_2_O_7_ and Sc_2_Ge_2_O_7_. Moreover, depending on the synthesis method, different structural modifications of Ln_2_Ge_2_O_7_ can be obtained. By conventional solid state synthesis, Ho_2_Ge_2_O_7_ has the tetragonal structure. However, the cubic phase of Ho_2_Ge_2_O_7_ can be synthesized by the high-pressure and high-temperature (HPHT) method. Therefore, pressure is an important weapon in a researcher's arsenal for exploring phase space. Pressure is also used to drive materials into new electronic states. Under high pressure, some materials become superconductors, others undergo magnetic phase transitions, and others undergo metal–insulator phase transitions.^20,21^ In magnetic pyrochlore oxides, pressure has been shown to freeze the spin-liquid ground state of Tb_2_Ti_2_O_7_.^22^ So, studies on the stability of Ho_2_Ge_2_O_7_ under high pressure are particularly important to understand its exotic magnetic phenomenon.

In this work, we successfully synthesized the two types of Ho_2_Ge_2_O_7_ using different methods. The structural stabilities of these two polymorphs of Ho_2_Ge_2_O_7_ were investigated by angle-dispersive synchrotron X-ray powder diffraction (ADXRD) at high pressures. Pressure-induced amorphization was found in the tetragonal Ho_2_Ge_2_O_7_. Meanwhile, the cubic Ho_2_Ge_2_O_7_ was stable up to the highest pressure tested.

## 2. Experimental section

### Synthesis

The tetragonal Ho_2_Ge_2_O_7_ was synthesized by standard solid state reaction method. High purity oxides of Ho_2_O_3_ (99.99%, powder) and GeO_2_ (99.99%, powder) were used as the starting materials. The raw materials with nominal compositions of Ho_2_Ge_2_O_7_ were uniformly mixed in an agate mortar. The powder obtained was pressed into small pellets and then calcined at 1373 K in air for 12 h. The cubic Ho_2_Ge_2_O_7_ was synthesized by the HPHT method. The as-prepared powders were loaded into a cubic anvil HPHT apparatus (SPD-6 × 600) at a temperature of 1573 K and a pressure of 5.2 GPa with a holding time of 15 min.

### Characterization

Under ambient conditions, the crystal phase structures of the synthesized samples were characterized by X-ray powder diffraction (XRD) using a Rigaku D/max-2500 with Cu Kα radiation (*λ* = 1.54056 Å) in the range 2*θ* from 10° to 90° at a scanning rate of 4° min^−1^. The high-pressure angle-dispersive XRD patterns for the two types of Ho_2_Ge_2_O_7_ were collected at beamline 4W2 of the Beijing Synchrotron Radiation Facility, using a monochromatic wavelength of 0.6199 Å. A diamond-anvil cell (DAC) was utilized to generate high pressure, using a T301 stainless steel gasket which was pre-indented to 50 μm thickness. One piece of the as-prepared samples, a small piece of ruby as the pressure calibrant^23^ and a 16 : 3 : 1 methanol/ethanol/water mixture as pressure-transmitting medium were loaded into the diamond-anvil cells. The experimental parameters, including the distance between sample and detector, were calibrated using CeO_2_ standard reference material. FIT2D software was employed to convert the image plate records into intensity *versus* diffraction angle 2*θ* patterns. Rietveld analyses were performed with the software GSAS.^24^ The refinement parameters were the lattice constants, the atomic position of oxygen, a Chebyshev polynomial background, pseudo-Voigt profile parameters, a common isotropic thermal parameter for all atom sites and an overall intensity scaling factor.

## 3. Results and discussion

### Crystal structures under ambient conditions

**Fig. 1:**
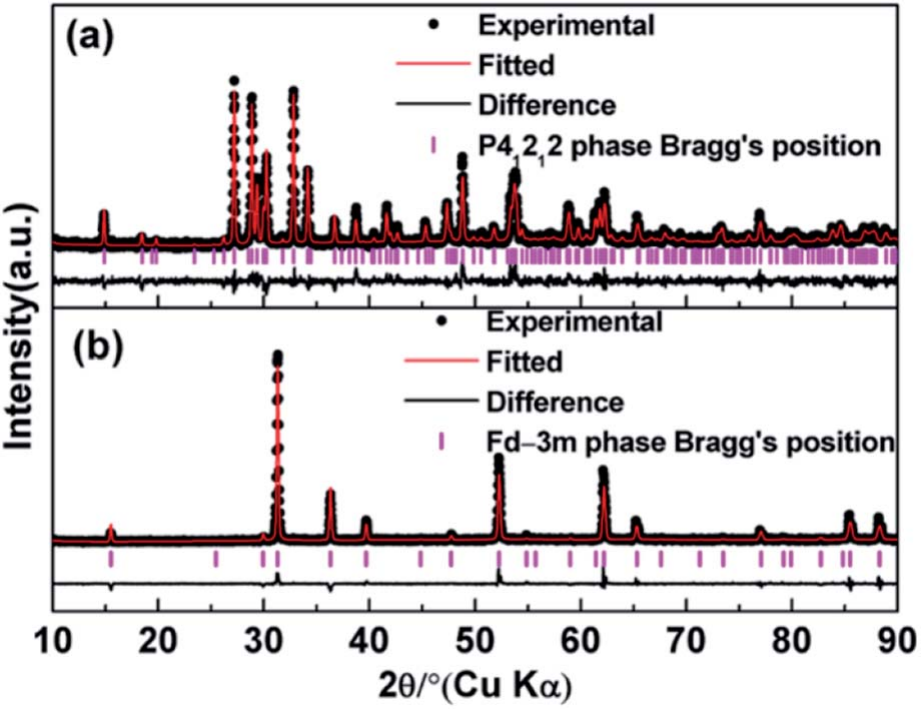
Observed, calculated and difference X-ray powder patterns of Ho_2_Ge_2_O_7_ at ambient pressure: (a) Rietveld refinement for the tetragonal Ho_2_Ge_2_O_7_ and (b) Rietveld refinement for the cubic Ho_2_Ge_2_O_7_.

**Fig. 2:**
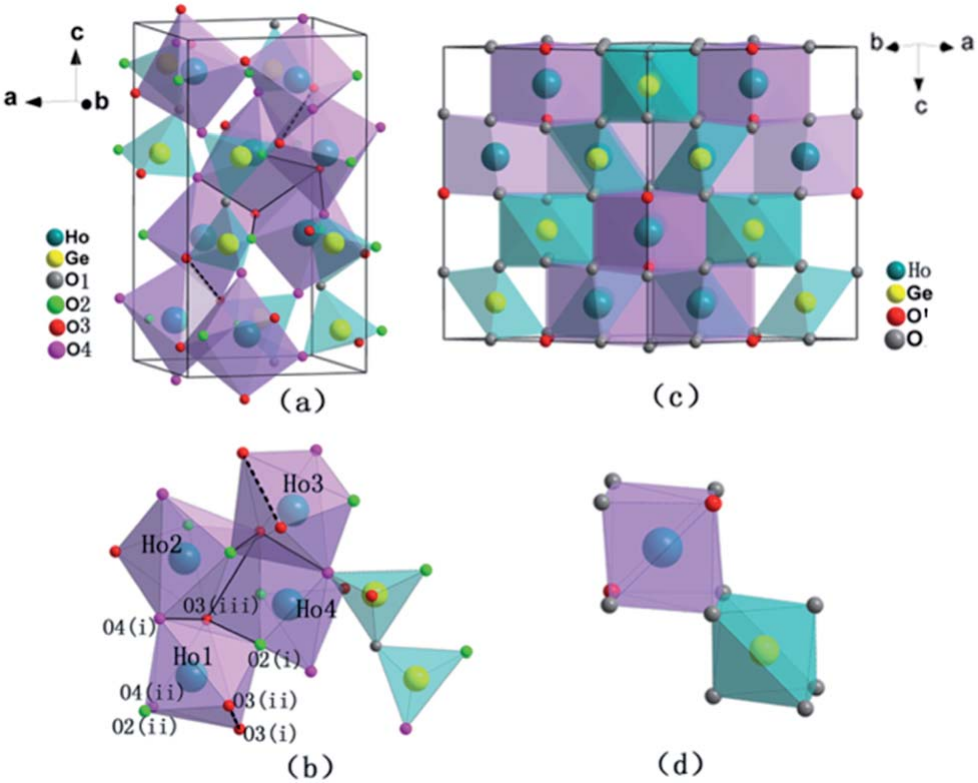
Schematic representation of the crystal structures of the tetragonal Ho_2_Ge_2_O_7_ ((a) and (b)) and the cubic Ho_2_Ge_2_O_7_ ((c) and (d)).

The observed and calculated XRD patterns of the two types of Ho_2_Ge_2_O_7_ together with their differences are shown in Fig. 1. The diffraction peaks in Fig. 1a match well with the tetragonal structure Ho_2_Ge_2_O_7_ (S.G. *P*4_1_2_1_2, no. 92) and the obtained cell parameters are: *a* = *b* = 6.8041(1) Å, *c* = 12.3734(1) Å, *V* = 572.83(1) Å^3^ with *Z* = 4. In this kind of crystal structure, each Ho^3+^ ion is coordinated to seven oxygen atoms. The Ge_2_O_7_ unit consists of two tetrahedra (GeO_4_) joined by a bridging oxygen atom. The bridging oxygen atoms of the Ge_2_O_7_ unit do not coordinate to the Ho^3+^ ion. The coordination polyhedron of the Ho^3+^ ion is a distorted pentagonal bipyramid with the Ho^3+^ ion located nearly in the basal plane. The pentagonal axis is almost parallel to the crystal *c*-axis. Each HoO_7_ polyhedron shares three of its O–O edges with neighboring polyhedra. Schematic illustrations are shown in Fig. 2a and b.

The crystal structure data of the cubic Ho_2_Ge_2_O_7_ were refined by Rietveld analysis of the X-ray powder diffraction data. The observed and calculated XRD patterns along with the difference plot are shown in Fig. 1b. The cubic Ho_2_Ge_2_O_7_ belongs to the *Fd*3̄*m* (no. 227) space group with the lattice parameters *a* = 9.8974(3) Å and *Z* = 8. In this cubic phase, it can be formulated as Ho_2_Ge_2_O_6_O′ with the Ge ion site at 16c, Ho at 16d, O at 48f and O′ at 8b. The Ho site (16d) coordination polyhedron is a distorted cube that generally contains larger cations, and the Ge site (16c) is a distorted octahedron. It is worth noting that there is only one adjustable positional parameter *x* for the O atom at the 48f site. Schematic illustrations of the cubic Ho_2_Ge_2_O_7_ are shown in Fig. 2c and d. In addition, the refined atomic position coordinates of the two polymorphs of Ho_2_Ge_2_O_7_ are given in Table 1.

**Table 1:** The refined atomic coordinates of the cubic and tetragonal Ho_2_Ge_2_O_7_ at ambient pressure

Compound	Ho_2_Ge_2_O_7_	Ho_2_Ge_2_O_7_
Crystal system	Cubic	Tetragonal
Space group	*Fd*3̄*m* (227)	*P*4_1_2_1_2 (92)
*a*/Å	9.8974(3)	6.8041(1)
*b*/Å	9.8974(3)	6.8041(1)
*c*/Å	9.8974(3)	12.3734(1)
Atoms	Wyckoff (*x y z*)	Wyckoff (*x y z*)
Ho	16d (0.5 0.5 0.5)	8a (0.8761(7) 0.3413(8) 0.1352(1))
Ge	16c (0 0 0)	8a (0.8857(3) 0.1469(9) 0.6199(4))
O(1)	48f (0.3255(10) 0.125 0.125)	4a (0.8141(5) 0.1859(6) 0.7500(8))
O(2)	8b (0.375 0.375 0.375)	8a (−0.0319(8) 0.1362(6) 0.6260(2))
O(3)		8a (0.0565(6) 0.3575(4) 0.5886(7))
O(4)		8a (0.6774(3) 0.1254(6) 0.5528(4))
Residuals^a^/%	*R*_wp_: 7.07%	*R*_wp_: 5.83%
	*R*_p_: 5.64%	*R*_p_: 4.61%

a *R*_wp_ and *R*_p_ as defined in GSAS.^24^

### Pressure-induced amorphization of the tetragonal Ho_2_Ge_2_O_7_

**Fig. 3:**
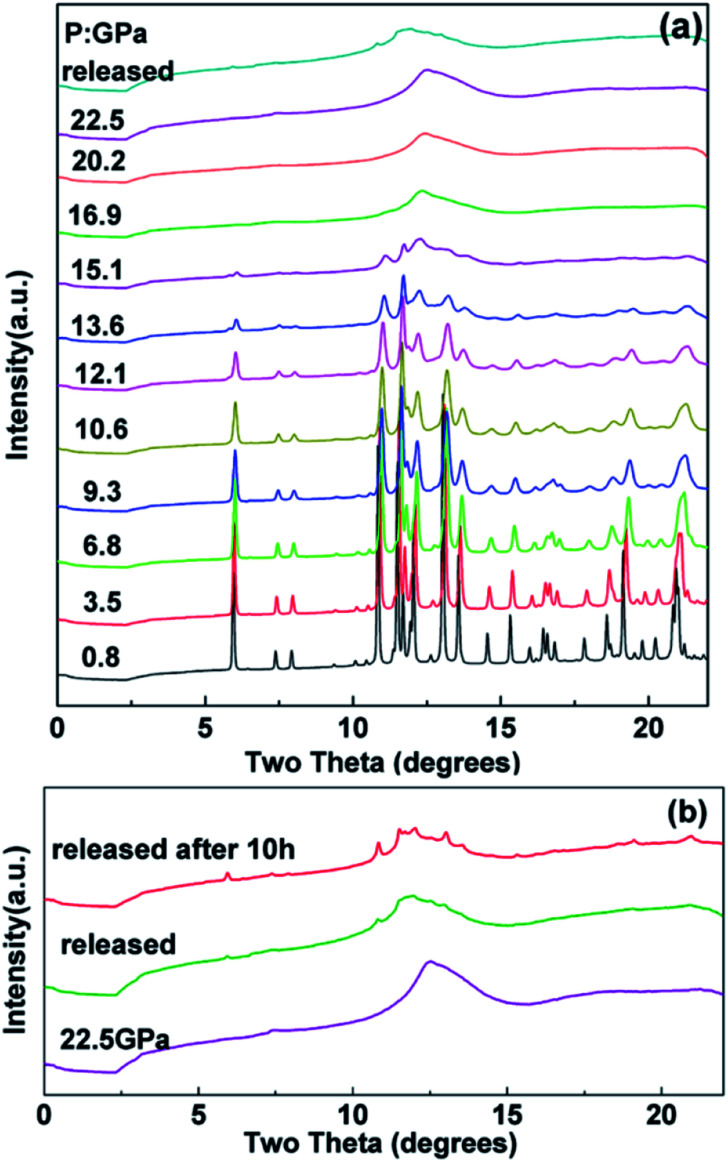
(a) Representative X-ray diffraction patterns of the tetragonal Ho_2_Ge_2_O_7_ at various pressures. (b) X-ray diffraction pattern for Ho_2_Ge_2_O_7_ after the release of pressure.

The *in situ* XRD patterns of the tetragonal Ho_2_Ge_2_O_7_ at various pressures up to 22.5 GPa were collected and a few representative patterns are shown in Fig. 3. As can be seen from Fig. 3a, the pressure-dependent X-ray data do not reveal any new diffraction peaks or peak splitting, which indicates that a typical tetragonal structure remains from ambient pressure up to 13.6 GPa. With further increasing pressure, most of the sharp bands have disappeared at 16.9 GPa and no diffraction peaks can be observed, which suggests the formation of an amorphous phase. After releasing the pressure, pressure-induced amorphization of Ho_2_Ge_2_O_7_ is maintained, which indicates the nonreversible nature of the phase transition. A few small peaks appeared after quenching and holding the sample under ambient conditions for 10 h. The recorded pattern for 10 h after release of pressure is almost identical to that of the original phase of tetragonal Ho_2_Ge_2_O_7_, as shown in Fig. 3b.

**Fig. 4:**
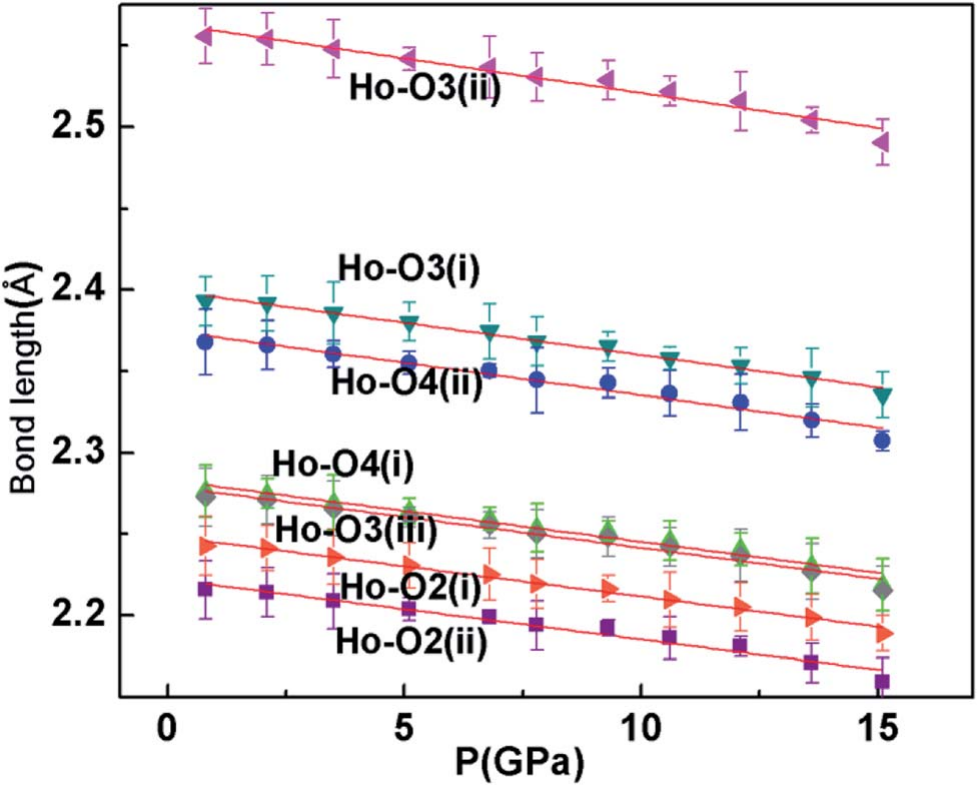
The Ho–O bond lengths in the tetragonal Ho_2_Ge_2_O_7_ at various pressures.

Pressure-induced amorphization has been the subject of intense study for the past few years because of its importance in materials science and solid state physics.^25–27^ A pressure-induced amorphization of α-NaVO_3_ was observed by Raman spectroscopy, which involved the tetrahedral VO_4_ chains breaking up abruptly at the transition pressure.^28^ In addition, silicates and metavanadate compounds also have chain structures and are the best examples of amorphous materials.^29–31^ As can be seen in Fig. 2a and b, each HoO_7_ polyhedron shares three of its O–O edges with neighboring polyhedra. And we can clearly see the four polyhedra (Ho_1_O_7_, Ho_2_O_7_, Ho_3_O_7_, Ho_4_O_7_) close together. The Ho_1_O_7_ polyhedron and Ho_3_O_7_ polyhedron share an O3(i)–O3(ii) edge with the four other closest polyhedra. In the crystal structure of Ho_2_Ge_2_O_7_, there is a chain, whose constituent unit (Ho_4_O_20_) is four edge-shared polyhedrons closely linked. In order to understand the reason for the amorphization, we plotted the variation of bond length with pressure for tetragonal Ho_2_Ge_2_O_7_ (Fig. 4). The bond lengths of Ho–O3(i) and Ho–O3(ii) always remain longer than the other Ho–O bond distance, from ambient pressure to the highest pressure. So the infinite Ho_4_O_20_ chain is relatively easy to disconnect at this junction. Pressure-induced amorphization of Ho_2_Ge_2_O_7_ is suggested to be associated with the breaking-up of long chains of the edge-shared polyhedron group Ho_4_O_20_. The tetragonal Ho_2_Ge_2_O_7_ showed long-range order at low magnetic fields, but its behavior was similar to spin-ice freezing in a sufficiently strong magnetic field.^32^ Owing to the appearance of pressure-induced amorphization, we can speculate that the tetragonal Ho_2_Ge_2_O_7_ may become paramagnetic under high pressure.

**Fig. 5:**
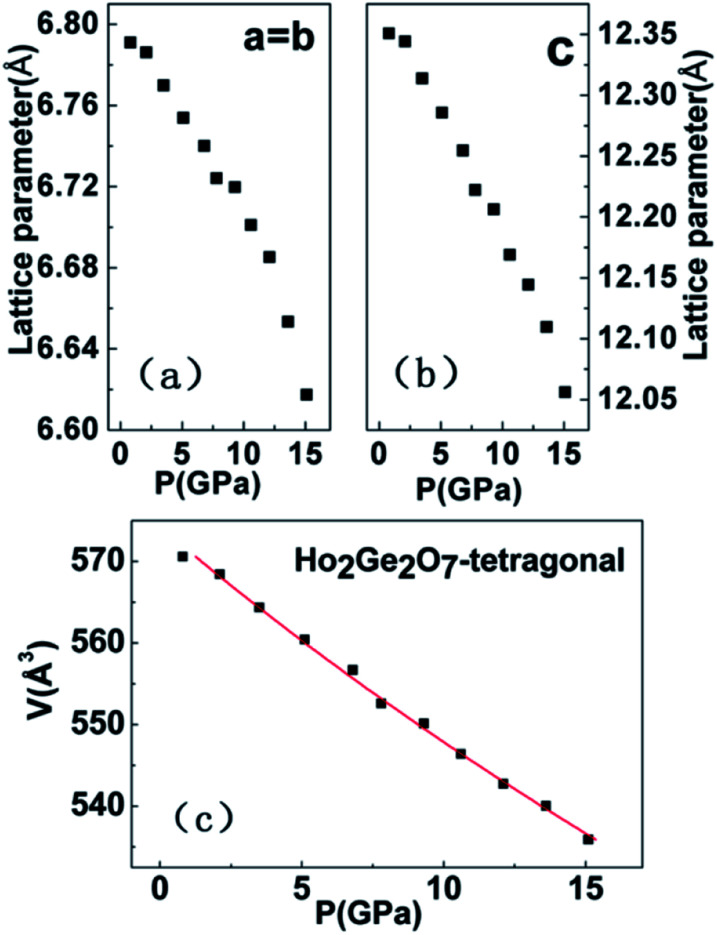
The lattice parameters ((a) and (b)) and unit cell volume (c) as a function of pressure for the tetragonal Ho_2_Ge_2_O_7_.

The pressure dependences of the lattice parameters and volume of tetragonal Ho_2_Ge_2_O_7_ up to 22.5 GPa are shown in Fig. 5. It is shown that the lattice parameters and volume of Ho_2_Ge_2_O_7_ decrease smoothly with increasing pressure. In order to determine the bulk modulus *B*_0_, its pressure derivative 
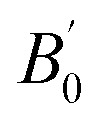
, and the molar volume under ambient conditions *V*_0_, the experimental pressure–volume data of the Ho_2_Ge_2_O_7_ were fitted to a third-order Birch–Murnaghan equation of state (Fig. 5c).^33^ The obtained bulk modulus *B*_0_ is 193(4) GPa for the tetragonal phase with fixed 
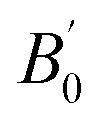
 = 4.

### Structural stability of the cubic Ho_2_Ge_2_O_7_ under high pressure

**Fig. 6:**
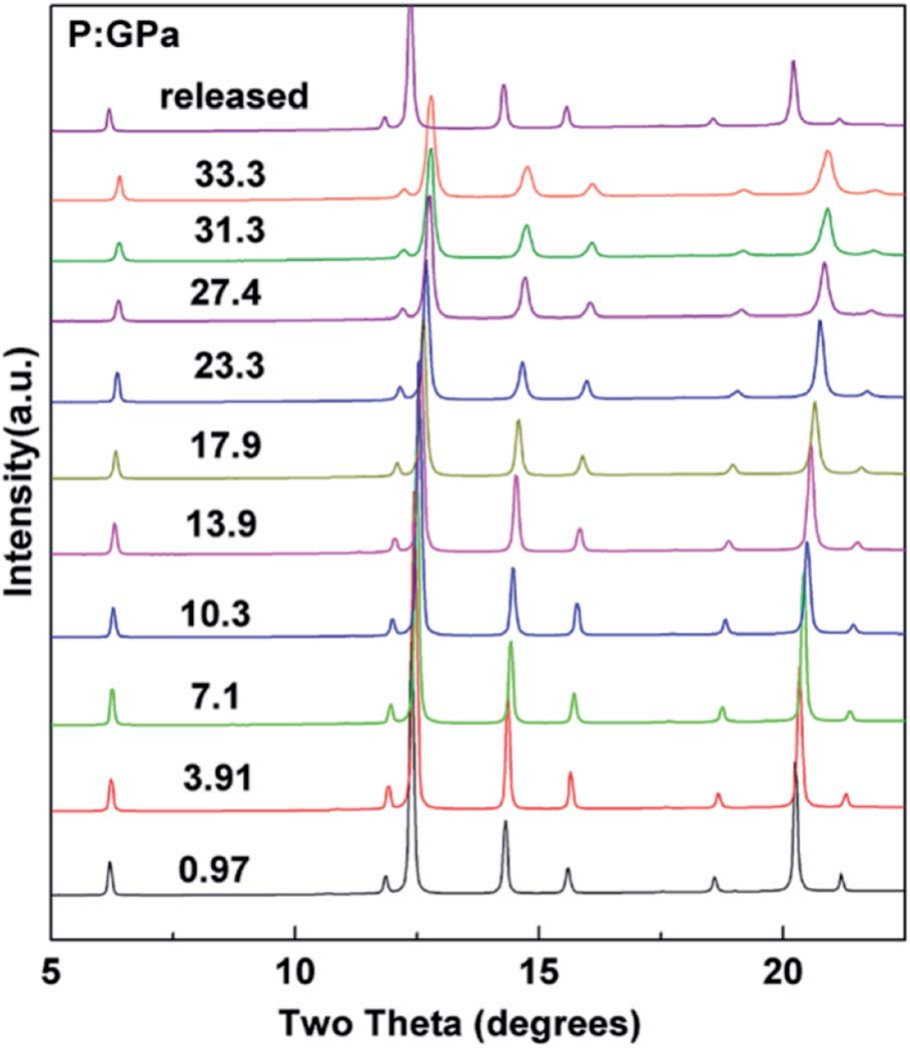
Selected XRD patterns of the cubic Ho_2_Ge_2_O_7_ with increasing pressure. The cubic structure is stable up to 33.3 GPa.

**Fig. 7:**
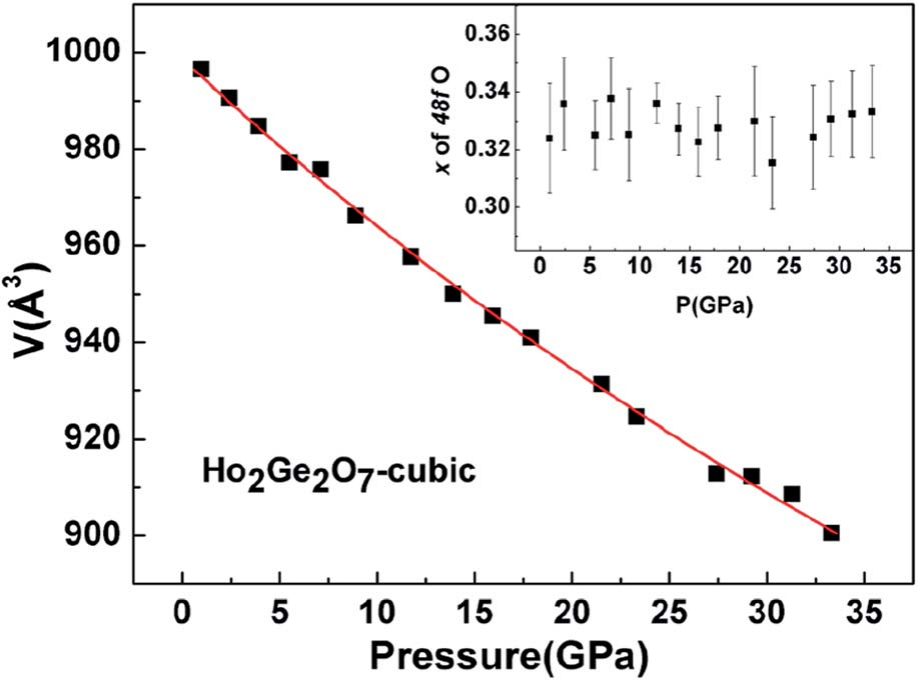
Observed *P*–*V* variation fitted with the third-order Birch–Murghnan (B–M) equation of state for the cubic Ho_2_Ge_2_O_7_. The inset shows the pressure dependence of the *x* parameter for the O_48f_ atom.

*In situ* XRD patterns of the cubic Ho_2_Ge_2_O_7_ were collected up to 33.3 GPa at room temperature and the representative patterns are shown in Fig. 6. No splitting or extra peaks appear in the patterns, demonstrating that the cubic phase of the Ho_2_Ge_2_O_7_ remains stable within the whole pressure range. In this ordered pyrochlore structure, all of the atoms are sited at defined positions, except for the O_48f_ atom. Hence, the degree of structural ordering can be determined by varying the *x* positional parameter of the O_48f_ atom. Previous studies on pyrochlore oxides have revealed that a sudden change of the *x* positional parameter of the O_48f_ atom occurred in the process of pressure-induced structural phase transition. For example, there was a rapid decrease of the *x* positional parameter with increasing pressure in Gd_2_Zr_2_O_7_, implying a phase transition;^34^ the *x*-coordinate of the O_48f_ atom increased dramatically after the transformation in Sm_2_Zr_2_O_7_.^35^ From our refined results, the *x* parameter for the O_48f_ atom as a function of pressure, as shown in the inset of Fig. 7, does not exhibit any anomaly up to the highest pressure employed, indicating the structural stability of the cubic Ho_2_Ge_2_O_7_. Recent experimental studies have reported that the pyrochlore Ho_2_Ge_2_O_7_ exhibited all the distinctive properties of a dipolar spin ice: a small, positive Curie–Weiss constant; Pauling zero-point entropy; magnetization which saturated to half the magnetic moment; a spin-freezing transition in the ac susceptibility; and the characteristic magnetic diffuse scattering of spin ices.^19^ The stability of the structure ensures the stability of these excellent properties under high pressure.

The pressure dependence of the unit cell volume of the cubic Ho_2_Ge_2_O_7_ is shown in Fig. 7. The data are fitted by a third-order Birch–Murnaghan equation of state as is the case for the tetragonal Ho_2_Ge_2_O_7_. The obtained bulk modulus *B*_0_ is 263(4) GPa, quite a bit larger than for other ordered pyrochlore oxide materials. For example, the bulk moduli of A_2_Ti_2_O_7_ (A = Ho, Y, Tb, Sm) were 213(2), 204(3), 199(1) and 164.8(1.5) GPa, respectively.^36,37^ And also for Gd_2_Zr_2_O_7_, the bulk modulus was 186(12) GPa.^34^ By comparison, the hardness of the cubic phase in Ho_2_Ge_2_O_7_ is higher than that of the tetragonal phase. The difference in bulk modulus might be due to the differences in structure and cohesive energy among these germanates.

## 4. Conclusions

In summary, the structural behaviors of two types of Ho_2_Ge_2_O_7_ were studied under high pressure by *in situ* XRD measurements. Pressure-induced amorphization was found in the tetragonal Ho_2_Ge_2_O_7_, which has a chain structure, and is suggested to be associated with the breaking-up of long chains of the edge-shared polyhedron group Ho_4_O_20_. By contrast, the cubic Ho_2_Ge_2_O_7_ structure is stable at high pressures up to 33.3 GPa. The bulk modulus of the tetragonal Ho_2_Ge_2_O_7_ was obtained as *B*_0_ = 193(4) GPa, and *B*_0_ = 263(4) GPa was obtained for the cubic phase.

## Conflicts of interest

There are no conflicts to declare.

## Supplementary Material

## References

[cit1] Bramwell S. T., Gingras M. J. P. (2001). Science.

[cit2] Gardner J. S., Gingras M. J. P., Greedan J. E. (2010). Rev. Mod. Phys..

[cit3] Castelnovo C., Moessner R., Sondhi S. L. (2012). Annu. Rev. Condens. Matter Phys..

[cit4] Gingras M. J. P., McClarty P. A. (2014). Rep. Prog. Phys..

[cit5] Bramwell S. T., Harris M. J., den Hertog B. C., Gingras M. J. P., Gardner J. S., McMorrow D. F., Wildes A. R., Cornelius A. L., Champion J. D. M., Melko R. G., Fennell T. (2001). Phys. Rev. Lett..

[cit6] Harris M. J., Bramwell S. T., McMorrow D. F., Zeiske T., Godfrey K. W. (1997). Phys. Rev. Lett..

[cit7] Ramirez A. P., Hayashi A., Cava R. J., Siddharthan R., Shastry B. S. (1999). Nature.

[cit8] Fennell T., Petrenko O. A., Fåk B., Bramwell S. T., Enjalran M., Yavors’kii T., Gingras M. J. P., Melko R. G., Balakrishnan G. (2004). Phys. Rev. B: Condens. Matter Mater. Phys..

[cit9] Kadowaki H., Ishii Y., Matsuhira K., Hinatsu Y. (2002). Phys. Rev. B: Condens. Matter Mater. Phys..

[cit10] Matsuhira K., Hinatsu Y., Tenya K., Amitsuka H., Sakakibara T. (2002). J. Phys. Soc. Jpn..

[cit11] Zhou H. D., Cheng J. G., Hallas A. M., Wiebe C. R., Li G., Balicas L., Zhou J. S., Goodenough J. B., Gardner J. S., Choi E. S. (2012). Phys. Rev. Lett..

[cit12] Zhou H. D., Bramwell S. T., Cheng J. G., Wiebe C. R., Li G., Balicas L., Bloxsom J. A., Silverstein H. J., Zhou J. S., Goodenough J. B., Gardner J. S. (2011). Nat. Commun..

[cit13] Castelnovo C., Moessner R., Sondhi S. L. (2008). Nature.

[cit14] Bramwell S. T., Giblin S. R., Calder S., Aldus R., Prabhakaran D., Fennell T. (2009). Nature.

[cit15] Jaubert L. D. C., Holdsworth P. C. W. (2009). Nat. Phys..

[cit16] Morris D. J. P., Tennant D. A., Grigera S. A., Klemke B., Castelnovo C., Moessner R., Czternasty C., Meissner M., Rule K. C., Hoffmann J.-U., Kiefer K., Gerischer S., Slobinsky D., Perry R. S. (2009). Science.

[cit17] Fennell T., Deen P. P., Wildes A. R., Schmalzl K., Prabhakaran D., Boothroyd A. T., Aldus R. J., McMorrow D. F., Bramwell S. T. (2009). Science.

[cit18] Giblin S. R., Bramwell S. T., Holdsworth P. C. W., Prabhakaran D., Terry I. (2011). Nat. Phys..

[cit19] Hallas A. M., Paddison J. A. M., Silverstein H. J., Goodwin A. L., Stewart J. R., Wildes A. R., Cheng J. G., Zhou J. S., Goodenough J. B., Choi E. S., Ehlers G., Gardner J. S., Wiebe C. R., Zhou H. D. (2012). Phys. Rev. B: Condens. Matter Mater. Phys..

[cit20] Deemyad S., Schilling J. S. (2003). Phys. Rev. Lett..

[cit21] Gavriliuk A. G., Struzhkin V. V., Lyubutin I. S., Ovchinnikov S. G., Hu M. Y., Chow P. (2008). Phys. Rev. B: Condens. Matter Mater. Phys..

[cit22] Mirebeau I., Goncharenko I. N., Cadavez-Pares P., Bramwell S. T., Gingras M. J. P., Gardner J. S. (2002). Nature.

[cit23] Mao H. K., Xu J., Bell P. M. (1986). J. Geophys. Res..

[cit24] Larson A. C. and Von Dreele R. B., General Structure Analysis System, LANSCE, MS-H805, Los Alamos, New Mexico, 1994

[cit25] Ponyatovsky E. G., Barkalov O. I. (1992). Mater. Sci. Rep..

[cit26] Kolobov A. V., Haines J., Pradel A., Ribes M., Fons P., Tominaga J., Steimer C., Aquilanti G., Pascarelli S. (2007). Appl. Phys. Lett..

[cit27] Yan X. Q., Li W. J., Goto T., Chen M. W. (2006). Appl. Phys. Lett..

[cit28] Shen Z. X., Ong C. W., Tang S. H., Kuok M. H. (1994). J. Phys. Chem. Solids.

[cit29] McNeil L. E., Grimsditch M. (1992). Phys. Rev. Lett..

[cit30] Mead C., Hemley R. J., Mao H. K. (1992). Phys. Rev. Lett..

[cit31] Li Y., Tang R., Li N., Li H., Zhao X., Zhu P., Wang X. (2015). J. Appl. Phys..

[cit32] Morosan E., Fleitman J. A., Huang Q., Lynn J. W., Chen Y., Ke X., Dahlberg M. L., Schiffer P., Craley C. R., Cava R. J. (2008). Phys. Rev. B: Condens. Matter Mater. Phys..

[cit33] Birch F. (1978). J. Geophys. Res..

[cit34] Zhang F. X., Lian J., Becker U., Ewing R. C., Hu J., Saxena S. K. (2007). Phys. Rev. B: Condens. Matter Mater. Phys..

[cit35] Zhang F. X., Lian J., Becker U., Wang L. W., Hu J., Saxena S. K., Ewing R. C. (2007). Chem. Phys. Lett..

[cit36] Scott P. R., Midgley A., Musaev O., Muthu D., Singh S., Surya-narayanan R., Revcolevschi A., Sood A., Kruger M. (2011). High Pressure Res..

[cit37] Zhang F. X., Manoun B., Saxena S. K., Zha C. S. (2005). Appl. Phys. Lett..

